# Clinical evaluation of two AI models for automated breast cancer plan generation

**DOI:** 10.1186/s13014-022-01993-9

**Published:** 2022-02-05

**Authors:** Esther Kneepkens, Nienke Bakx, Maurice van der Sangen, Jacqueline Theuws, Peter-Paul van der Toorn, Dorien Rijkaart, Jorien van der Leer, Thérèse van Nunen, Els Hagelaar, Hanneke Bluemink, Coen Hurkmans

**Affiliations:** Department of Radiation Oncology, Catharina Hospital, Eindhoven, The Netherlands

**Keywords:** Deep learning, U-net, Breast cancer, Dose mimicking, cARF, Autoplanning

## Abstract

**Background:**

Artificial intelligence (AI) shows great potential to streamline the treatment planning process. However, its clinical adoption is slow due to the limited number of clinical evaluation studies and because often, the translation of the predicted dose distribution to a deliverable plan is lacking. This study evaluates two different, deliverable AI plans in terms of their clinical acceptability based on quantitative parameters and qualitative evaluation by four radiation oncologists.

**Methods:**

For 20 left-sided node-negative breast cancer patients, treated with a prescribed dose of 40.05 Gy, using tangential beam intensity modulated radiotherapy, two model-based treatment plans were evaluated against the corresponding manual plan. The two models used were an in-house developed U-net model and a vendor-developed contextual atlas regression forest model (cARF). Radiation oncologists evaluated the clinical acceptability of each blinded plan and ranked plans according to preference. Furthermore, a comparison with the manual plan was made based on dose volume histogram parameters, clinical evaluation criteria and preparation time.

**Results:**

The U-net model resulted in a higher average and maximum dose to the PTV (median difference 0.37 Gy and 0.47 Gy respectively) and a slightly higher mean heart dose (MHD) (0.01 Gy). The cARF model led to higher average and maximum doses to the PTV (0.30 and 0.39 Gy respectively) and a slightly higher MHD (0.02 Gy) and mean lung dose (MLD, 0.04 Gy). The maximum MHD/MLD difference was ≤ 0.5 Gy for both AI plans. Regardless of these dose differences, 90–95% of the AI plans were considered clinically acceptable versus 90% of the manual plans. Preferences varied between the radiation oncologists. Plan preparation time was comparable between the U-net model and the manual plan (287 s vs 253 s) while the cARF model took longer (471 s). When only considering user interaction, plan generation time was 121 s for the cARF model and 137 s for the U-net model.

**Conclusions:**

Two AI models were used to generate deliverable plans for breast cancer patients, in a time-efficient manner, requiring minimal user interaction. Although the AI plans resulted in slightly higher doses overall, radiation oncologists considered 90–95% of the AI plans clinically acceptable.

**Supplementary Information:**

The online version contains supplementary material available at 10.1186/s13014-022-01993-9.

## Background

Whole breast radiotherapy is a widely accepted local treatment for early breast cancer after breast-conserving surgery, as it reduces local recurrence and breast cancer death [1]. However, the process of treatment planning is manual and iterative, which can be time consuming. Moreover, plan quality is prone to differences in experience of the planner [2]. In recent years, several methods have been developed to automate this process, including machine learning (ML) and deep learning (DL) approaches [3–5]. Although most studies focus on the treatment sites prostate or head and neck, the number of studies focusing on whole breast radiotherapy increases [6–9]. Most of the ML and DL approaches result in a dose distribution prediction per voxel, which is not directly clinically applicable. Inverse optimization or dose mimicking can be used to infer clinically deliverable plans [10–12]. To evaluate the automatically generated plans, many quantitative metrics are reported, such as mean and maximum doses to organs, and dose differences compared to clinical plans. However, to validate the usefulness of the plans in the clinical workflow, additional qualitative review is recommended [5, 13]. In this study, two previously developed ML and DL models for whole breast radiotherapy are evaluated in a blinded review procedure by four physicians, in addition to quantitative review.

## Methods

### Patients

20 patients with left-sided node-negative breast cancer, treated in the Catharina Hospital between July 2020 and January 2021, were included in the study. The research was conducted on anonymized patient data according to Dutch data protection and privacy legislation.

As patients were treated in moderate deep inspiration breath hold, all treatment plans were made on breath hold CT scans (3 mm slice thickness). Clinical target volume (CTV) and organs at risk (OAR) were contoured following the ESTRO guidelines [14]. The planning target volume (PTV) was generated by 5 mm expansion of the CTV, followed by 5 mm cropping under the skin. The average PTV volume was 890 ± 425 cm^3^.

### Treatment plans

Patients were treated with a prescribed total dose of 40.05 Gy in 15 fractions, using tangential beam intensity modulated radiotherapy (IMRT) plans with beam energy of either 6 (n = 17) or 10 MV (n = 3), depending on patient anatomy. For both manual and AI planning, each tangential beam consisted of at least one open segment and together, the two tangential beam directions had up to 8 segments of at least 9 cm^2^. The dose calculation grid resolution was 3 mm isotropically. RayStation Treatment Planning System (TPS) 9B (RaySearch Medical Laboratories, Stockholm, Sweden) was used for manual treatment planning, while Research version 9B (build 8.99) was used for the AI plans. Both TPS versions use a collapsed cone convolution dose calculation algorithm (type b) [15]. The manual and the AI plans were calculated on the same hardware (NVIDIA RTX6000, 12 vCPU, 64 GB RAM).

The isocenter was positioned in the center of the PTV, unless this would lead to a collision with the gantry, in which case the isocenter was moved inwards. The tangential beams were initialized at 130 and 310 degrees and subsequently, automatic beam angle optimization was performed in the 3D-CRT module of the TPS aimed at minimizing the dose to the heart, lungs and contralateral breast as previously described by Bakx et al. [16]. Using the same initial beam setup and beam energy, three plans were made: a manual plan and two model-based plans. The clinical goals used are summarized in Table 1.

**Table 1:** Percentage of the plans that met the clinical goals for all three planning methods

	Clinical goals met [%]		
	Manual	cARF	U-net
PTV D2% < 107%	100	90	95
MHD < 3 Gy	95	95	95
MLD < 6 Gy	100	100	100
*Contralateral breast, mean dose* < *1 Gy*	100	100	100
*External -PTV, Max 10 cm*^*3*^ < *107%*	95	95	95
*MHD* < *2 Gy*	95	90	90
*Lungs V5Gy* < *50%*	100	95	95
*MLD* < *4 Gy*	100	100	100

The goals in italics are of lower priority than the others, meaning they are target values, not hard constraints

MHD, mean heart dose; MLD, mean lung dose (sum of both lungs)

#### Manual plans

Manual plans were made by radiotherapy technologists (RTTs), both more and less experienced, following routine clinical practice, using an inverse planning technique in which the RTT’s chose the beam energy and adjusted the objective functions*.* While all clinical goals should be met, the initial focus was on achieving the PTV coverage and additionally, the planner attempted to reduce the MHD and MLD while maintaining the PTV coverage goal. The planners were asked to focus on the task at hand and note the time required. After a first round of optimization, the leaves of the tangential fields with a contribution of > 100 MU per fraction are retracted from the skin surface (~ 4 cm) to promote robustness to swelling and breath hold position, followed by further optimization. All plans were scaled to ensure that 98% of the PTV volume received at least 95% of the prescribed dose.

#### AI planning

For the same patients, the RayLearner module of RayStation 9B was used to generate two additional plans using two separate AI planning models. In both cases, the models were used to predict the dose distribution based on the patient anatomy. As the predicted dose distributions are not directly clinically applicable, dose mimicking is used to translate them to deliverable plans afterwards. The dose mimicking algorithm available in the TPS is used, which involves direct machine parameter optimization to approximate the predicted dose distribution, while taking dose constraints into account (settings listed in Additional file 1: Table A.1). To obtain the final dose distribution, three intermediate collapsed cone convolution dose calculations were performed, ending with a final collapsed cone convolution dose calculation [12].

The first model is an in-house developed model, which is an adapted version of the U-net architecture of Nguyen [17]. Input of the model consists of contours for the PTV, the body, heart and lungs. The second model was developed by RaySearch and is based on contextual atlas regression forests (cARF)[10]. A more detailed description of both models and their training on in-house clinical data was previously published by Bakx et al. [7] and can also be found in the Additional file 1. The current study focused on the clinical applicability of both models.

After the AI plan generation, the leaves of the tangential fields with a contribution of > 100 MU per fraction were retracted from the skin surface (~ 4 cm) by the RTT and one last optimization run (40 iterations) was performed. As a final step, all plans were scaled to ensure that 98% of the PTV volume received at least 95% of the prescribed dose. The time required to perform the various parts of the AI planning process was recorded. Since the AI plan generation required no actions by the planner, except manually opening the leaves of the tangential fields, no influence of the planner’s experience was expected. The plans were generated by two planners trained in using the RayLearner module. The optimization time and time for manual actions were monitored separately.

### Plan evaluation

Plans were evaluated based on a set of predefined DVH parameters, on conformity using the Paddick conformity index (CI = $\frac{{\left({V}_{PTV} \cap { V}_{100\%Iso}\right)}^{2}}{{V}_{PTV} \times {V}_{100\%Iso}}$) [18], number of monitor units (MU) and time required for the planning procedure. The DVH parameters were chosen in line with the Dutch national evaluation parameters for breast cancer treatment plans and are listed in Table 1 [19]. Besides, a complexity metric was calculated to compare created segments of manual and AI plans [20]. Additionally, a more subjective analysis was performed by 4 radiation oncologists, all specialized in breast cancer radiotherapy. The radiation oncologists were asked to independently perform a blind comparison of the three plans for all patients. They judged whether the separate plans were clinically acceptable and ranked them based on their preference, allowing equal ranking in case of no preference, resulting in a ranking score of 1 (highest preference) to 3 (lowest preference). They were encouraged to provide reasons for their choice.

Statistical evaluations were performed in IBM SPSS Statistics Version 25. For all comparisons, the Wilcoxon Signed Rank test was used and a *p* value of 0.05 or lower was considered statistically significant. Unless stated otherwise, the *p* values demonstrate whether the specific AI plan is different from the manual plan.

## Results

### Dose distribution and clinical goals

**Fig. 1:**
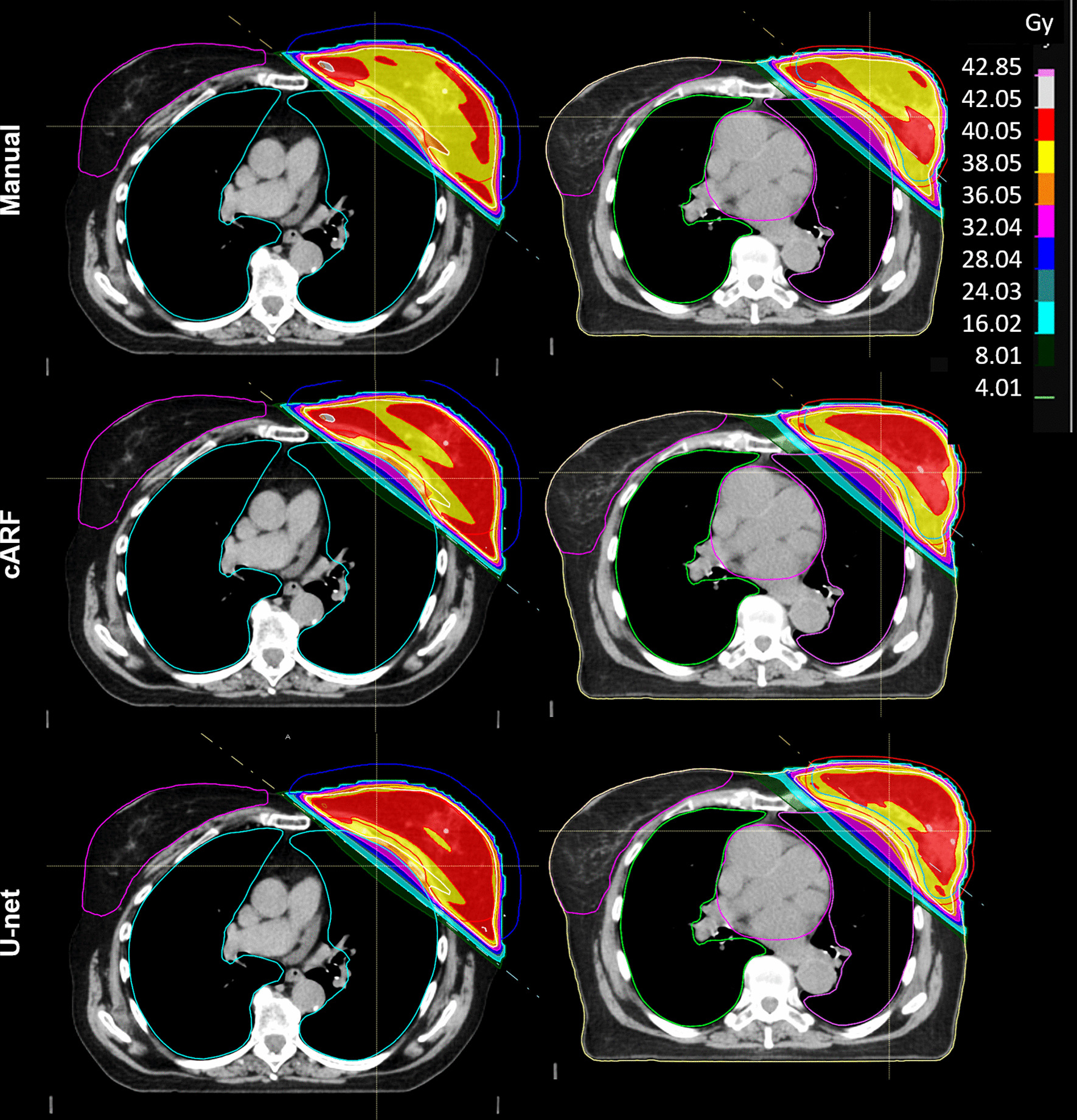
Examples of an axial slice of the dose distribution of the different plans for two patients

Examples of the dose distribution for the different plans are shown in Fig. 1. The percentage of the plans in which particular clinical goals were met is stated in Table 1. The PTV D2% goal was not met in 2/20 cARF plans, compared to 1/20 U-net and none of the manual plans. For one patient, none of the plans met the MHD goal (manual plan 4.10 Gy; cARF 3.76 Gy; U-net 4.47 Gy).

**Fig. 2:**
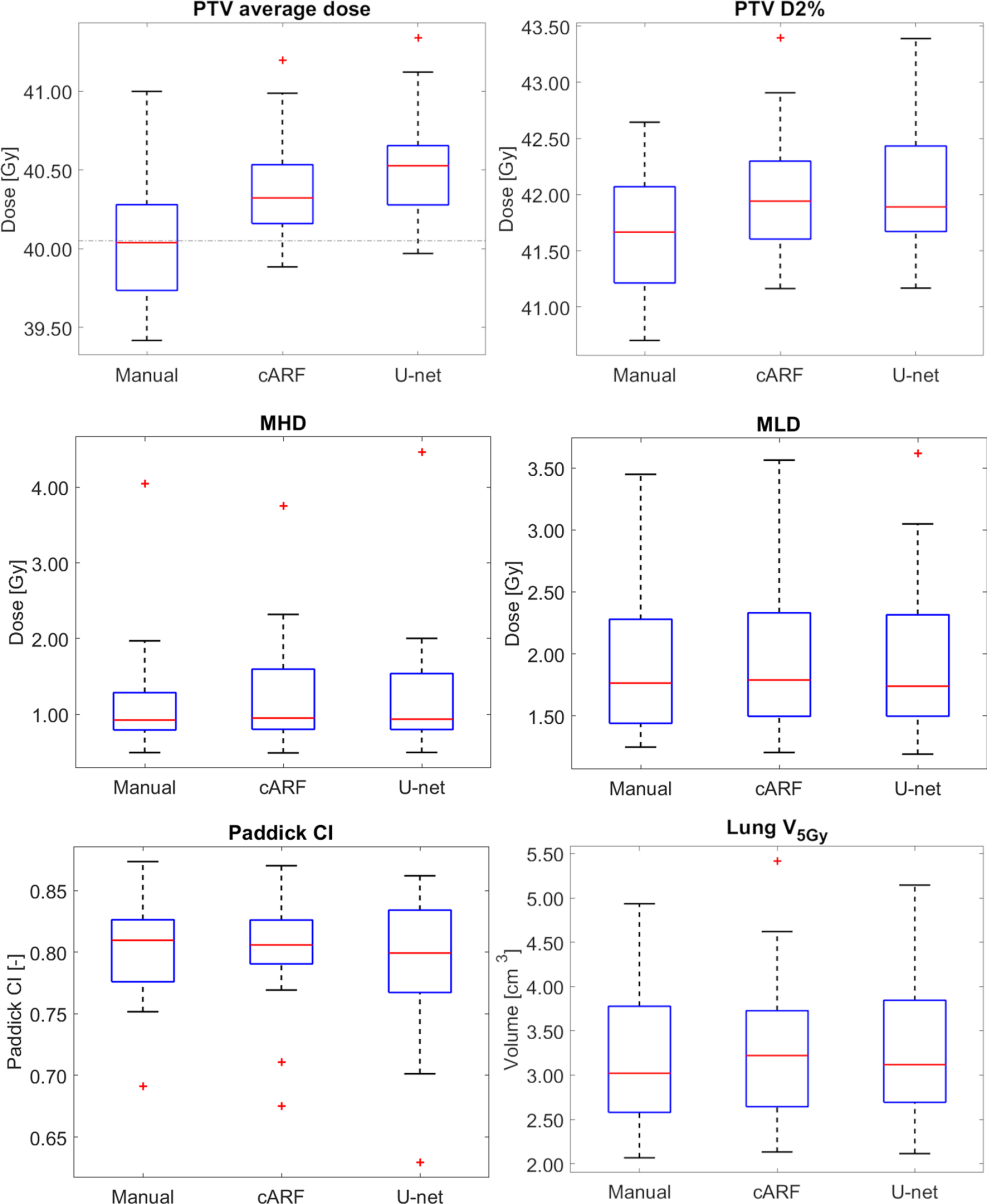
Relevant DVH-parameters for PTV, heart and lung. The red crosses represent outliers. The median is indicated with a red line. For the PTV, the dotted line represents the prescribed dose of 40.05 Gy

Relevant DVH-parameters are displayed in Fig. 2 and Table 2. After scaling the dose to ensure PTV V95% = 98%, both the average and the D2% PTV dose of cARF and U-net plans were higher than that of the corresponding manual plan. The median difference in PTV average dose was + 0.30 Gy (range − 0.01 to 0.83 Gy, *p* < 0.01) for the cARF plans and + 0.37 Gy for the U-net plans (range − 0.08 to 1.07 Gy, *p* < 0.01), respectively. The median difference in PTV D2% was + 0.39 Gy (range − 0.04 to 1.32 Gy, *p* < 0.01) for the cARF plans and + 0.47 Gy for the U-net plans (range − 0.45 to 1.19 Gy, *p* < 0.01), respectively. The Paddick CI was not significantly different (Fig. 2). Additional DVH parameters are reported in Additional file 1: Table A.2.

**Table 2:** DVH-parameters for PTV, heart and lungs represented as mean dose ± standard deviation

	PTV			Lungs		Heart
	Average dose [Gy]	Difference w.r.t. prescribed dose [%]	D2% [Gy]	MLD [Gy]	V5Gy [cm^3^]	MHD [Gy]
Clinical	40.07 ± 0.40	+ 0.1	41.69 ± 0.57	1.92 ± 0.62	325 ± 85	1.17 ± 0.77
cARF	40.37 ± 0.35*	+ 0.8	42.01 ± 0.56*	1.98 ± 0.64*	333 ± 87*	1.23 ± 0.76*
U-net	40.53 ± 0.36*	+ 1.2	42.02 ± 0.56*	1.96 ± 0.64	331 ± 86	1.24 ± 0.87*

Asterisks indicate significant differences with the manual dose distribution (*p* ≤ 0.05)

For the cARF plan, mean heart and lung doses were higher than in the corresponding manual plan, albeit slightly (MHD: median difference + 0.02 Gy, range − 0.29 to 0.49 Gy, *p* < 0.05; MLD: median difference + 0.04 Gy, range − 0.09 to 0.42 Gy, *p* < 0.05; Lung V5Gy median difference + 0.06 Gy, range − 0.13 to 0.48 Gy). For the U-net plan, the MHD was higher than for the manual plan (median difference + 0.01 Gy, range − 0.2 to 0.37 Gy, *p* < 0.05).

The number of MU required for the cARF plans was higher than for the manual plans (median + 7%, *p* < 0.05). There was no difference in the number of MU needed for the U-net compared with the manual plans. Also, no significant difference was found between the complexity of the manual and U-net plans (median 0.52, range 0.34–0.72 and 0.57, range 0.45–0.79, respectively), whereas the complexity of the cARF plans (median 0.61, range 0.48–1.04) was significantly higher.

### Plan generation time

**Fig. 3:**
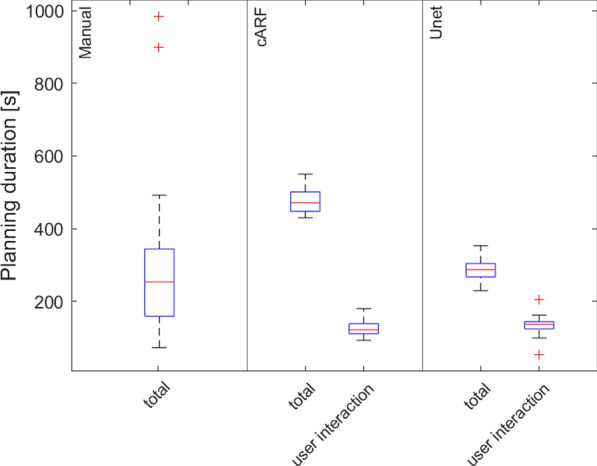
Time needed for plan generation. For the AI plans, the time spent on user interaction is separately specified. The red crosses represent outliers

The time needed to generate a plan is reported in Fig. 3. The median time needed was 253 s (range 72–984 s) for the manual plans, 471 s (430–550 s, *p* = 0.014) for the cARF plans and 287 s (229–353 s, *p* = 0.411) for the U-net plans. The variation in plan generation time is larger for the manual plans than for both AI plans. After subtracting the computation time, the remaining time needed for user interaction was 121 s (92–180 s) for the cARF plans and 136 s (53–205 s) for the U-net plans. For the manual plans, the computation time was not recorded separately as it is often interleaved with manual adjustments.

### Evaluation by radiation oncologists

**Fig. 4:**
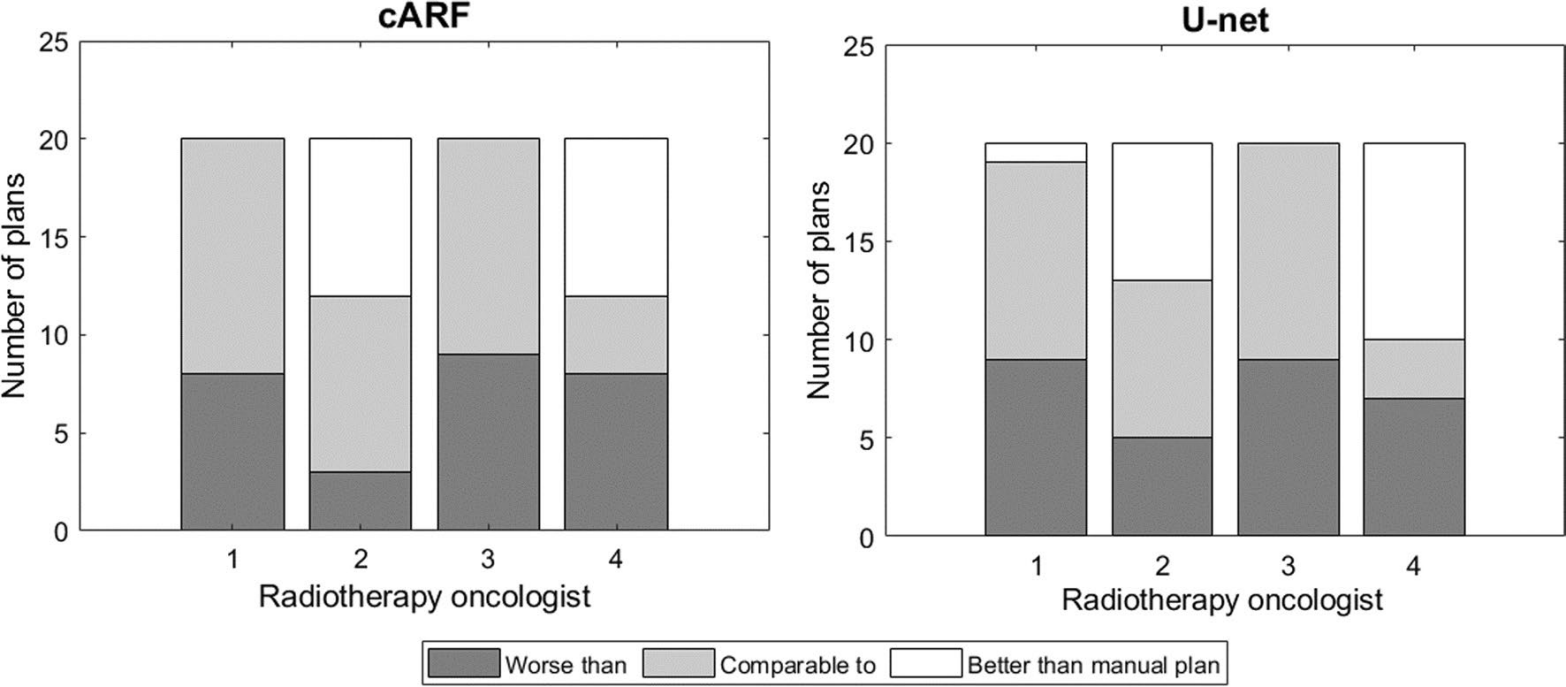
Ranking of the AI plans in comparison with the manual plan by the Radiation Oncologists on an individual basis

The results of the evaluation of the plans by the radiation oncologists are summarized in Table 3 and Fig. 4. Individual scoring results can be found in the Additional file 1: Figure A.2. 90–95% of the AI plans were considered clinically acceptable. The radiation oncologists had a slight preference for the manual plans, as can be deduced from the lower average rank. In 35% of the cases the 4 observers independently agreed that the AI plan was equally suitable or better than the manual plan. In 15 and 20% of the cases (for cARF and U-net respectively), the AI plan was considered worse than the manual plan by all radiation oncologists. In 45–50% of cases there was no consensus.

**Table 3:** Evaluation of the plans by the radiation oncologists

	Acceptable for all	Average ranking	Consensus autoplan worse	Consensus autoplan equal or better	No consensus
	[%]	–	[%]	[%]	[%]
Manual	90	1.4			
cARF	90	1.7	15	35	50
U-net	95	1.6	20	35	45

The appreciation of the AI plans differed between radiation oncologists, as can be seen from Fig. 4. One of the radiation oncologists always preferred the manual plan (observer 3), one preferred the U-net plan over the manual plan for a single patient (observer 1) and the other two radiation oncologists preferred the AI plan relatively often (observers 2 and 4). Based on the explanations provided, the radiation oncologists focused mostly on dose coverage in the PTV. Interestingly, the two radiation oncologists who preferred the AI plans relatively often (observer 2 and 4), praised the coverage of the 100% isodose line, while observer 3 favored a 95% isodose coverage and never preferred any AI plan over the manual plan. Other factors underlying their choices were the absence of hotspots (as visually perceived) and lower mean heart doses. Judging from Fig. 4 and the average rank given to both AI plans, both models performed comparably.

## Discussion

In this study, two previously developed dose prediction models for whole breast radiotherapy were clinically validated. In addition to a quantitative review of DVH parameters, a qualitative review was performed by four physicians through a blinded review of manually and automatically created plans.

Both AI plans resulted in a significant higher average and maximum dose to the PTV and higher average dose to the heart, whereas only the cARF model resulted in plans with a significant higher dose to the lungs. For the U-net plan, the higher dose to the PTV compared to the manual plan was also observed previously for the mimicked dose distributions [7], although, this was not the case for the cARF plans. Furthermore, the difference in dose to PTV could partially be explained by the fact that an extra criterion for the average PTV dose was introduced in our institute, based on the Dutch national consensus, after training of the models (Additional file 1: Table A.3). While the planners were using the old criteria for all manual plans, their recent experience with slightly stricter PTV dose criteria could have inadvertently influenced their work. However, the differences in doses to the PTV and OARs were not found to be clinically relevant, which is reflected by the high acceptance rate for both models.

For the automated planning process, the mimicked dose distributions were evaluated without further optimization. In 90 to 95% of the cases, the AI models produced clinically acceptable plans, leading to an efficient and consistent workflow. In cases were the plans are not assessed as clinically acceptable, the TPS allows for further manual optimization and this is the way we intend to introduce these AI models clinically. Additionally, the mimick settings can be optimized to better adhere to the clinician’s preferences or to small adjustments in clinical goals without having to retrain the model. Also, multiple plans can be generated with mimick settings focusing more on specific clinical goals. Future improvements in plan quality could therefore include optimization of the mimick settings.

Time reduction is an important goal of automation of the treatment planning process. In the manual planning process, patients with an aberrant anatomy lead to an increase of time spent on optimization of the treatment plan, resulting in the outliers which are visualized in Fig. 3. However, the time spent by both models is independent on patient anatomy and therefore results in a more consistent, predictable process. The AI plans corresponding to the two manual plans that took ≥ 15 min to make, were considered clinically acceptable by all radiation oncologists, while for one patient, the manual plan was rejected by one radiation oncologist. As the computation time for the AI plans is dependent on hardware, it is more relevant to analyze the user interaction time. As is shown in Fig. 3, the user interaction time is lower for both models, than the total time spent when manually creating a treatment plan. Additional scripting of the remaining manual tasks could reduce the interaction time even further. Taking this into account, next to the fact that hardware for computing will only improve in the near future, it can be stated that both models result in a more time efficient process. Based on the slightly higher time efficiency and lower plan complexity metric than the cARF model, we plan to introduce the U-net model into clinical practice, where we will of course adhere to e.g., the Medical Device Regulation.

A few other studies involving dose prediction models for breast cancer have been performed. Ahn et al*.* compared an in-house developed DL model, based on U-Net, with the auto-planning module available in the treatment planning system Eclipse [6]. For the PTV they found differences between both models of less than 1%, but larger differences were found for the OARs, resulting in better prediction of the DL model. However, the plans predicted by the DL model were not executable and still need an extra step to be clinically deliverable. Hedden and Xu compared a two-dimensional (2D) and three-dimensional (3D) model, both based on the U-Net architecture, where they found better results for the 3D model [8]. Dose differences of the mean dose for all regions were within 0.05%, except outliers, where the 3D model outperformed the 2D model for the right lung and heart. Similar to Ahn et al*.*, the predicted doses still need inverse planning to be clinically deliverable, and are currently intended to be used as reference during the planning process. In contrast to these two studies, Sheng et al*.* developed a ML model able to create clinically deliverable plans, using a random forest model for fluence estimation, and enabling interactive planning by a fluence fine tuning model [9]. Except for an increased mean heart dose for the AI plans, no statistically significant differences were found between the AI plans and clinical plans.

A limitation of the above mentioned studies is the lack of a qualitative review, which is highly recommended to validate clinical usefulness [5, 13]. Recently, McIntosh et al*.* published a study about the clinical integration of an AI model for prostate cancer, including quantitative and qualitative review [21]. In two phases, a retrospective simulation and a prospective deployment study phase, 89% of plans generated by the AI model were deemed to be clinically acceptable, which is comparable to our results. Overall, the AI-generated plan was selected in 72% of cases, although notable differences in the reviewer’s preference for manual or AI plans were observed. In our study, this difference in preference is reflected in the results, as two radiation oncologists almost never preferred the AI plans, while the two other radiation oncologists often preferred them. However, in 35% of the cases there was a consensus that the AI plan was equal to or better than the manual plan. The observed lack of consensus could be considered a result of differing personal preferences, which calls for further education, harmonization and guidelines.

## Conclusions

In summary, two AI models were used to generate deliverable and clinically acceptable plans for left-sided, node-negative breast cancer patients, requiring minimal user interaction. The radiation oncologists considered 90–95% of the AI plans clinically acceptable and plan generation was time-efficient. Therefore, we plan to introduce the U-net model-based plan into clinical practice. Future improvements will entail optimization of the dose mimicking settings and expanding the AI toolbox with models for node-positive breast cancer patients.

## Supplementary Information

**Additional file 1.** Content: more elaborate model description and additional figures and tables as referenced to in the main manuscript text.

## Data Availability

The datasets used and/or analysed during the current study are available from the corresponding author on reasonable request.

## Abbreviations

2D: Two dimensional
3D: Three dimensional
AI: Artificial intelligence
cARF: Contextual atlas regression forests
CI: Conformity index
CT: Computed tomography
CTV: Clinical target volume
DL: Deep learning
DVH: Dose volume histogram
IMRT: Intensity modulated radiotherapy
MHD: Mean heart dose
ML: Machine learning
MLD: Mean lung dose
MU: Monitor unit
OAR: Organ at risk
PTV: Planning target volume
RTT: Radiotherapy technologist
TPS: Treatment planning system
