# Supplementary material for: Clinical evaluation of two AI models for automated breast cancer plan generation
Source: Radiat Oncol. 2022 Feb 5;17:25. doi: 10.1186/s13014-022-01993-9 (PMC8817521; doi:10.1186/s13014-022-01993-9)
Supplement: Supplementary file 1 — Additional file 1. Content: more elaborate model description and additional figures and tables as referenced to in the main manuscript text. [file 13014_2022_1993_MOESM1_ESM.docx]

# Supplementary information

| **ROI** | **DVH-parameter** | | | | **Weight** |
| --- | --- | --- | --- | --- | --- |
| cARF | | | | | |
| PTV | D98% | > | 38.05 | Gy | 5 |
|  | D2% | < | 42.85 | Gy | 5 |
| Heart | MHD | < | 3.00 | Gy | 1 |
| Lungs | MLD | < | 6.00 | Gy | 1 |
| External | D_max_ | < | 42.85 | Gy | 1 |
|  |  |  |  |  |  |
| U-net | | | | | |
| PTV | D98% | > | 38.05 | Gy | 5 |
|  | D_max_ | < | 42.50 | Gy | 5 |
| External | D_max_ | < | 43.00 | Gy | 1 |

**Table A. 1.** Mimick settings for cARF and U-net model.

*AI planning*

For generating the AI plans, two separate AI planning models were used in this study. The first model, an in-house developed model, is an adapted version of the U-net architecture, based on the architecture used by Nguyen et al. [1]. The architecture is visualized in Figure A.1. The input of the model contains four channels, representing four contours; PTV, heart, lungs and the external contour. These contours are represented by binary masks, except for the PTV mask, where the voxels inside the contour were assigned a weight value, equal to the prescribed dose.

The second model was based on a contextual atlas regression forest (cARF), and is extensively described by McIntosh and Purdie [1, 2]. The method consists of two phases; (1) the atlas-to-image mapping phase, and (2) the atlas-selection phase. In the first phase, the relationship between image features and the dose distribution are modeled by training atlas regression forests (ARFs). In the second phase a model is trained to select the most relevant ARF for a new patient, using density estimation over the observed image features. To find the most probable dose distributions, a conditional random field model is used.

[1] Nguyen D, Long T, Jia X, Lu W, Gu X, Iqbal Z, Jian S. A feasibility study for predicting optimal radiation therapy dose distributions of prostate cancer patients from patient anatomy using deep learning. Scientific reports 2019;9:1–10. <https://doi.org/10.1038/s41598-018-37741-x>

[2] McIntosh C and Purdie TG, Contextual atlas regression forests: multiple-atlas-based automated dose prediction in radiation therapy. IEEE transactions on medical imaging 2015;35:1000–12. <https://doi.org/10.1109/tmi.2015.2505188>

[3] McIntosh C and Purdie TG. Voxel-based dose prediction with multi-patient atlas selection for automated radiotherapy treatment planning. Physics in Medicine & Biology 2016;2:415. <https://doi.org/10.1088/1361-6560/62/2/415>


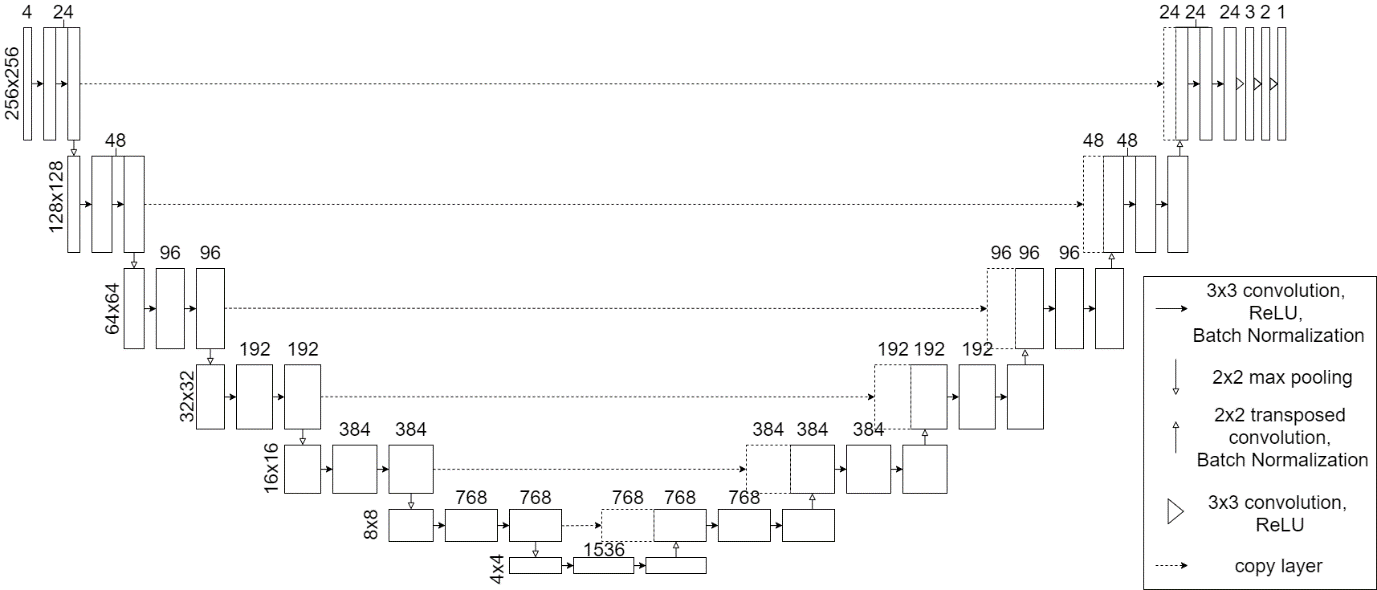


**Figure A. 1.** U-Net architecture used for the in-house developed model. Numbers on the side represent 2D feature size, numbers above layers represent number of features of that layer.

**Table A. 2.**  Additional DVH-parameters for the different types of plans. All values are reported as median (range).

|  | Clinical goals met | | |
| --- | --- | --- | --- |
|  |  | [%] |  |
|  | **Manual** | **cARF** | **U-net** |
| PTV average dose > 99% | 95 | 100 | 100 |
| PTV average dose < 101% | 90 | 70 | 50 |

**Table A. 3** Clinical goals added after model development based on recent Dutch consensus guidelines.

**
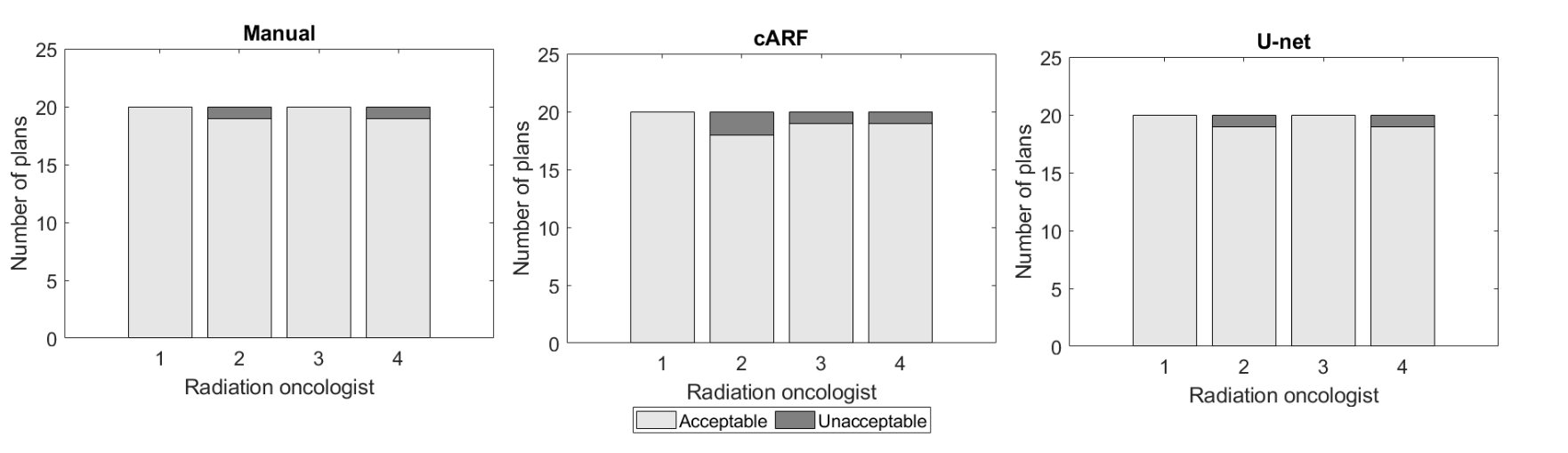
**

**Figure A. 2.** Judgement of the clinical acceptability of the plans by the Radiation Oncologists on an individual basis.
